# Associations between elementary and middle school teachers’ physical activity promoting practices and teacher- and school-level factors

**DOI:** 10.1186/s12966-021-01129-4

**Published:** 2021-05-19

**Authors:** Ann Pulling Kuhn, Edward Kim, Hannah G. Lane, Yan Wang, Rachel Deitch, Lindsey Turner, Erin R. Hager, Elizabeth A. Parker

**Affiliations:** 1 Growth and Nutrition Division, Department of Pediatrics, University of Maryland School of Medicine, 737 Lombard St, Baltimore, MD 21201 USA; 2 University of Maryland, Baltimore, Baltimore, MD 21201 USA; 3 Department of Population Health Sciences, Duke University School of Medicine, Durham, NC 27705 USA; 4 Department of Prevention and Community Health, George Washington University, 20052 Washington DC, USA; 5 Boise State University, College of Education, Boise, ID 83725 USA; 6 Department of Physical Therapy and Rehabilitation Science, University of Maryland School of Medicine, 21201 Baltimore, MD USA

**Keywords:** Teacher physical activity, School physical activity, Physical activity promotion

## Abstract

**Background:**

Few studies have evaluated teacher- and school-level characteristics associated with implementation of recommended physical activity (PA) promoting practices. The purpose of this study is to examine associations between teachers’ PA practices and: [1] teacher-level factors, including their own PA, and [2] school-level factors.

**Methods:**

This cross-sectional study examined time spent daily in light PA (LPA) and moderate-vigorous PA (MVPA) in association with 7 teacher PA practices among 288 classroom/special area teachers and teaching assistants in 20 urban, suburban and rural schools (recruited through a school wellness trial) in 4 districts. LPA and MVPA was assessed using 24-h ankle accelerometry (up to seven consecutive days). A sum score for teacher PA practices was assessed via survey (7 items; sum score range: 7–35; Cronbach’s alpha = 0.73; higher scores indicate more PA promoting practices). Teacher-level factors included gender, race, self-reported height/weight, years teaching, and education. School-level factors included school type, free-and-reduced-price meal eligibility, student racial/ethnic composition, and urbanicity. Analyses included multilevel regression models, accounting for clustering within schools and adjusting for demographic covariates and school district.

**Results:**

Teachers were 91% female, 63% elementary, 60% white, mean age 43.2 years (*SD* = 11.3), and 41% obese). Teachers wore accelerometers an average of 5.8 days, spent 399.6 min in LPA (*SD* = 85.0) per day, 24.1 min in MVPA (*SD* = 14.4) per day, and the mean teacher PA practices sum score was 22.4 (*SD* = 5.0). Every 15-min increase in MVPA was related to an increase in teacher PA practices sum score (coeff =1.07; *SE* = 0.28; *p* < 0.001). Female gender (versus males; coeff = − 1.95; *SE* = 0.92, *p* = 0.034), an obese weight status (versus non-obese; coeff = − 1.38; *SE* = 0.54, *p* = 0.010), and teaching in a middle school (versus elementary; coeff = − 3.86; *SE* = 0.54, *p* < 0.001) were associated with lower teacher PA practices scores. LPA was not associated with teacher PA promoting practices.

**Conclusions:**

Teachers with higher MVPA, but not higher LPA, and those without obesity were more likely to implement PA promoting practices that could positively impact their students’ PA. Similar to prior studies, these practices were more commonly implemented in elementary schools and by male teachers. Future studies in schools should explore whether improvement of teacher health behaviors subsequently impacts student health behaviors.

**Trial registration:**

Clinical Trials, NCT03432715; Registered on 02/2/2018.

**Supplementary Information:**

The online version contains supplementary material available at 10.1186/s12966-021-01129-4.

## Background

Physical activity is important for improving children and adolescents’ physical and mental health [1–3], and overall health-related quality of life [4, 5]. Since children spend a majority of their time in schools, the school environment is a logical setting to increase the amount of time that students spend in physical activity (PA) [6]. Children should obtain at least 30 min of the recommended daily 60 min of moderate-to-vigorous PA (MVPA) while at school [7]. However, a recent systematic review found that less than 25% of children and adolescents from 20 countries met this recommendation [8]. For this reason, the Centers for Disease Control (CDC) recommends a “whole of school” approach, called a Comprehensive School Physical Activity Program (CSPAP), in which students are provided with PA opportunities through physical education class, recess, classroom PA, before and after school programs, and community and staff involvement [9]. Such school-wide PA interventions have been effective at increasing children’s PA behaviors [10, 11].

One important aspect of school-wide PA programs is the role of the teacher in promoting and providing PA opportunities [12, 13]. Classroom teachers are essential stakeholders in creating PA promoting environments by providing PA opportunities to increase PA in and outside of the classroom [12, 14]. PA inside of the classroom can include incorporating PA into academic content, scheduling short PA breaks between academic content, or using PA as a transition between activities [15]. Also, policies that prohibit teachers from using PA as a punishment (e.g., pushups) in a classroom or physical education setting are recommended to increase student PA [15]. Numerous classroom-based PA programs have been developed such as Take 10! [16] or Energizers [17] and are effective at positively impacting children’s PA behaviors [18, 19] and cognitive function [20–22]. Additionally, PA role modeling by teachers can have a positive impact on student PA both inside and outside of the classroom [23]. While many schools report adopting classroom PA programs, the implementation of teacher PA promoting practices at those schools remains low, particularly in lower socioeconomic status (SES) schools.

Research has identified teacher- and school-level factors related to teachers’ PA promoting practices in schools [24]. Teacher-level factors may include teachers’ attitudes and beliefs about teacher PA promoting practices (e.g., perceived ease of implementation, motivation, and confidence/self-efficacy) [25–28]. Years of fulltime teaching experience may also be influential; but research findings are mixed [29, 30]. Additionally, a higher level of education is known to be associated with PA levels in general [31], however the relationship between level of education and teacher PA promoting practices has yet to be studied. An important teacher-level factor to examine is personal engagement in PA, as it may also positively influence student PA behaviors [23, 32]. Studies have found associations between teachers’ self-reported PA behaviors and personal PA-related beliefs with their willingness and competence for implementing PA promoting practices [14, 33, 34]; however, less is understood about what teacher-level factors relate to actual implementation of those practices.

School-level factors related to PA promoting practices in schools have also been identified, including the presence of district and administrative support, environmental resources, time, and implementation support for administration and teachers [18–21]. Other school-level factors that may affect PA opportunities for students include school type (elementary vs. secondary), school SES, school racial/ethnic composition, and school urbanicity [35, 36]. Elementary schools typically provide more PA opportunities than secondary schools, given secondary schools’ challenges with overcoming academic priorities and having PA opportunities that cater to older students [15]. Rural and low SES schools are often found to be under-resourced, and provide fewer PA opportunities [35]. Racial/ethnic disparities also exist indicating that majority nonwhite schools may be less likely to have PA conducive environments [36, 37].

Given that teachers’ PA promoting practices can positively impact student PA behaviors [14], it is critical to identify teacher and school-level factors that may be associated with teacher PA promoting practices. Furthermore, teacher-level factors (including, but not limited to, demographic characteristics and PA level) must be disentangled from school-level factors to ensure that interventions to improve teacher PA promoting practices and reducing PA disparities are tailored to address key factors. Therefore, the purpose of this study is to examine associations between teacher PA promoting practices and: (1) teacher-level factors (i.e., objectively measured daily average light PA [LPA] and MVPA, and individual demographics) and (2) school-level factors (i.e., school type, school percentage of students who are eligible for free and reduced meal services (FARMS; a measure for school SES), school racial/ethnic composition, and school urbanicity). We hypothesized that teachers who spent more time in daily average LPA and MVPA would report implementing more teacher PA promoting practices, while controlling for individual and school demographic variables. In addition, we hypothesized that other individual-level factors, such as teachers’ years of full-time experience and education level would positively predict teacher PA promoting practices.

## Methods

### Participants

Data collected from classroom and special area teachers (hereafter, “teachers”) and teaching assistants, who participated in the Wellness Champions for Change (WCC) study [38] were used in this study. The teachers were from 20 elementary and middle schools from four school systems/districts in a Mid-Atlantic state. WCC is a school-based cluster-randomized controlled trial examining the impact of school wellness policy implementation on student health behaviors. As part of this study, teachers were invited to participate in a baseline evaluation through lunch meetings hosted by WCC study staff, school staff meetings, e-mail contact, and printed advertisements. A total of 465 teachers in 20 schools were recruited for data collection. After providing written informed consent, teachers were emailed an electronic survey [Qualtrics Version 2017.11 (Provo, UT)]. Survey data on demographics and teacher PA promoting practices were used for the current analyses. A subset of these teachers (*n* = 325, 70%) were also offered the opportunity to wear an accelerometer to objectively measure PA, based on accelerometer inventory (a randomization program was used to randomly identify teachers to wear accelerometers, as there were a limited number of devices) and presence in the school on the day the accelerometers were distributed. Of the 325 teachers who received an accelerometer, 23 had incomplete or invalid accelerometry data and 14 were missing survey data, leading to a final sample size of 288 (88.6% of eligible sample). IRB approval was granted by the University of Maryland School of Medicine and school district IRBs in the four school systems/districts. Teachers received a $20 dollar gift card for participating.

### Measures

#### Outcome variable

##### Teacher PA promoting practices

Teacher PA promoting practices were assessed through an adapted Perceptions of the Environment at School (PEAS) survey [39]. The original version of the PEAS survey consists of 40 items that assess students’ perceptions of policies, physical environment, and practices related to healthy eating and PA at school, including 7 items focused specifically on PA in and outside of the classroom that reflected recommendations for implementing classroom- and school-based PA [40]. For teachers, questions were slightly adapted (e.g., “my teacher is a good role model for PA” to “I am a good role model for PA”). Items addressed the following: in-class student movement breaks (1 item), rewarding students with extra PA (1 item), punishing students with extra PA (1 item), role modeling by performing PA in front of students during the school day or talking about importance of PA (3 items), and beliefs about role modeling for PA (1 item). Of these, one item was reverse coded: “I have my students run laps, do push-ups or another PA if they misbehave in class.” Responses were on a five-point Likert-type scale ranging from 1 (never) to 5 (always), which were summed to create an overall score (Cronbach’s alpha = 0.73), with higher scores indicative of healthier practices (items listed in Table 2).

### Predictor variables

#### Teacher-level factors

##### Demographics and Body Mass Index (BMI)

Teachers self-reported demographic variables including: gender, race/ethnicity, age, level of education, and years of experience teaching. Teachers also self-reported height and weight, which was used to calculate BMI (kg/m^2^). Thresholds for overweight (25 kg/m^2^) and obesity (30 kg/m^2^) were applied to examine weight status.

##### Teacher PA

Teachers wore an Actical accelerometer (Philips Respironics, Bend, OR) to objectively measure PA. Acticals were placed on teachers’ non-dominant ankle with a non-removable, reinforced hospital band worn next to the skin under the sock [41]. Acticals were attached on the first day of data collection and removed approximately 7 days later (collecting activity data in 24-h periods). Accelerometer counts were collected in 15-s epochs. Actical software (version 2.12) was used to download the accelerometer data and smoothed to 1-min intervals. Data included average minutes of LPA and MVPA using thresholds validated among adolescents [41] and previously applied to adult populations [42].

#### School-level factors

Demographic data for each school was collected from the National Center for Education Statistics (NCES) or school websites and included: school type (i.e., elementary, middle), student eligibility for free and reduced-price meal services (FARMS), student racial/ethnic composition, and school urbanicity (i.e., rural, urban, suburban).

### Statistical analysis

Data were analyzed using SPSS version 26. To address missing data, hot deck imputation was utilized by replacing missing data values with observed values from other respondents by role and grade level taught within the same data set [43]. Means and standard deviations were calculated for continuous variables and frequencies were calculated for categorical variables. Bivariate analyses using mixed-effect models that accounted for school clustering determined associations between teacher PA promoting practices sum scores and LPA, MVPA, and each covariate. The covariates were selected to the multivariate model based on the significant relations with the outcome in the bivariate analyses for parsimony. For the main analyses, we examined LPA and MVPA by 60- and 15- min non-contiguous increments of time, respectively, to produce interpretable model coefficients [44]. Multilevel regression models were used to assess associations between the dependent variable (teacher PA promoting practices sum score) and independent variables (LPA and MVPA), with a random intercept at the school level to account for the clustering of teachers within each school [45]. An unconditional model that included the random intercept only was run for the teacher PA promoting practices sum score to assess the school clustering effect and the intraclass correlation coefficient (ICC) within schools. The second model only included independent variables at Level 1 (individual level: gender, weight status, LPA, MVPA), whereas the third model also included a school-level covariate at Level 2 (i.e., school type). Both models controlled for the number of days the accelerometer was worn.

## Results

Table 1 shows participant and school characteristics. The sample included 237 classroom and special area teachers and 51 teaching assistants. The majority taught in elementary schools (63%) and were non-Hispanic White race/ethnicity (61%). Nearly two thirds had a graduate degree (62%) and 73% had less than 20 years of teaching experience. The mean age of participants was 44 years and since it was highly correlated with years of experience (*r* = 0.6), was excluded from analyses. Sixty-five percent had a BMI classification of overweight/obese (based on self-reported height and weight). Of the 20 schools, 11 (55%) were elementary, 13 had < 75% FARMS eligibility, 14 had a student racial/ethnic composition of < 50% white students, and 11 were located in suburban areas. Based on independent t-tests for continuous variables and chi-square analyses for categorical variables, included teachers (*n* = 288) were more likely than excluded teachers (*n* = 37) to be white (*p* < 0.001), have less years of experience (*p* < 0.003), have a graduate degree (*p* = 0.004), and work at a school where < 75% of students were eligible for FARMS (*p* < 0.001) and work at a school with a school racial composition of > 50% non-Hispanic White race/ethnicity (*p* = 0.012).

**Table 1:** Descriptive characteristics (*N*/%)

Participant characteristics	Participant sample (*n* = 288)
Gender	
Male	27 (9.4)
Female	261 (90.6)
Type of teacher	
Classroom or special area teacher	237 (82.3)
Teaching assistant	51 (17.7)
Race/ethnicity	
Black or African American	75 (26.0)
Non-Hispanic White or Caucasian	175 (60.8)
Other^b^	28 (9.4)
Level of education	
No graduate degree	110 (38.2)
Masters or doctoral degree	178 (61.8)
Years of experience	
1 to 15 years	163 (56.6)
More than 15 years	125 (43.4)
Weight Status (self-reported height/weight, BMI calculated)^a^	
Normal weight	89 (30.9)
Overweight	71 (24.7)
Obese	117 (40.6)
School characteristics	School sample (*n* = 20)
Grade level	
Elementary	11 (55.0)
Middle school	9 (45.0)
Racial composition	
≥ 50% Non-Hispanic White	6 (30.0)
< 50% Non-Hispanic White	14 (70.0)
Free and reduced-price meal eligibility	
< 75% eligible	13 (65.0)
≥75% eligible	7 (35.0)
School urbanicity	
Rural	6 (30.0)
Suburban	11 (55.0)
Urban	3 (15.0)

*Note.* ^a^No participants were categorized as underweight for self-reported weight status

^b^Other category merged any race that composed less than 5% of the sample

Teachers’ daily time in LPA averaged 399.6 min (*SD* = 85.0; range 157–697), and daily MVPA averaged 24.1 min (*SD* = 14.4; range 0–57). The mean teacher PA promoting practices sum score was 22.4 (*SD* = 5.0; range: 11–34). LPA, MVPA, and teacher PA promoting practices sum scores had skewness and kurtosis values within ±1, indicating they did not deviate from normal distribution [46].

Table 2 shows percent of responses for each PEAS item reported by participants. Almost half of teachers reported giving their students short breaks for PA most of the time or always. Most teachers reported that they never use PA as punishment to their students (87%), but few reported giving their students extra PA time for good behavior most of the time or always (15%). Nearly one third reported talking about being physically active with their students most of the time or always (31%), and 33% reported never being active with them. About half reported telling students that it was important to be active most of the time or always (48%) and slightly less than half reported being a good role model for PA most of the time or always (42%).

**Table 2:** Percentage teacher PA promoting practices reported by participants

Teacher PA promoting practices (Cronbach’s alpha = 0.73)	Never (1)	Once in a while (2)	Sometimes (3)	Most of the time (4)	Always (5)
I give my students short breaks in class where they stand up or get out of their seats to move (like brain breaks or energizers)	6.3%	14.6%	29.5%	22.2%	27.4%
I have my students run laps, do push-ups or another PA if they misbehave in class^a^	86.8%	4.9%	6.3%	.7%	1.4%
I give my students extra PA time for being well behaved in class	36.1%	19.4%	28.8%	9.7%	5.9%
I talk about being physically active or playing sports in front of my students	9.7%	21.2%	37.8%	15.3%	16.0%
I play sports or do PA with my students during the school day	33.0%	29.5%	22.9%	6.3%	8.3%
I tell my students it is important to move and be active	8.7%	18.8%	24.7%	26.4%	21.5%
I am a good role model for PA	9.0%	19.4%	29.5%	25.7%	16.3%
Sum Score (Mean + SD):	22.43 + 5.00				

*Note.* ^a^Item reverse coded

Table 3 shows results of the bivariate analyses, accounting for clustering within schools, for differences in PA and teacher PA promoting practices sum scores by demographic variables. MVPA was higher among teachers who were male (*p* < 0.001), white (*p* = 0.004), of normal weight (*p* = 0.007) and worked in a school with a racial composition > 50% white students (*p* < 0.001), that was located in a rural area (*p* < 0.001). LPA was higher among teachers who were of normal weight (*p* = 0.006) and taught at the elementary level (*p* = 0.026). For teacher PA promoting practices, teachers had higher scores if they were male (*p* = 0.011), were of normal weight (*p* = 0.003), and taught at the elementary level (*p* < 0.001).

**Table 3:** Bivariate analysis with PA in minutes by LPA and MVPA

	LPA		MVPA		Teacher PA promoting practices sum score	
	Mean ± SD	Wald chi square (p)	Mean ± SD	Wald chi square (p)	Mean ± SD	Wald chi square (p)
Gender						
Male	404.4 ± 84.7	0.34 (0.558)	34.9 ± 16.0	19.42 (< 0.001)**	24.1 ± 6.2	6.47 (0.011)*
Female	399.1 ± 85.2		22.9 ± 13.8		22.3 ± 4.8	
Race						
White	405.7 ± 79.0	1.19 (0.275)	26.2 ± 14.5	8.52 (0.004)**	22.7 ± 5.2	0.09 (0.760)
Non-white	390.1 ± 93.2		20.8 ± 13.6		22.1 ± 4.7	
Weight Status						
Normal weight	416.7 ± 88.8	10.31 (0.006)**	27.6 ± 14.8	9.88 (0.007)**	23.8 ± 5.3	11.45 (0.003)**
Overweight	413.5 ± 73.7		25.0 ± 15.7		22.9 ± 4.5	
Obese	381.1 ± 85.7		21.1 ± 12.7		21.2 ± 4.9	
Education level						
No graduate degree	409.2 ± 88.2	2.61 (0.106)	23.9 ± 13.5	0.04 (0.848)	22.0 ± 4.8	1.05 (0.306)
Masters/doctoral degree	393.7 ± 82.8		24.2 ± 14.9		22.7 ± 5.1	
Years experience						
1 to 15 years	397.2 ± 84.9	0.06 (0.814)	23.8 ± 13.5	< 0.001 (0.966)	22.2 ± 4.8	0.01 (0.942)
More than 15 years	401.7 ± 85.4		24.5 ± 15.5		22.8 ± 5.3	
School type						
Elementary	409.7 ± 89.8	4.94 (0.026)*	24.6 ± 14.6	0.05 (0.830)	23.9 ± 4.5	48.48 (< 0.001)**
Middle	382.8 ± 73.9		23.3 ± 14.1		19.9 ± 4.7	
School Race – White						
50% or more	412.8 ± 74.6	2.86 (0.091)	27.8 ± 14.7	14.00 (< 0.001)**	23.4 ± 4.9	1.04 (0.308)
Less than 50%	390.5 ± 90.7		21.5 ± 13.6		21.8 ± 4.9	
School FARMS eligibility						
Less than 75%	400.85 ± 75.14	< 0.01 (0.961)	25.31 ± 14.64	3.70 (0.055)	22.36 ± 5.17	.50 (0.479)
75% or more	396.50 ± 106.08		21.13 ± 13.39		22.60 ± 4.57	
School urbanicity						
Suburban	386.8 ± 86.8	4.43 (0.109)	22.1 ± 13.4	15.36 (< 0.001)**	21.5 ± 5.1	2.17 (0.339)
Urban	408.1 ± 107.8		18.8 ± 14.4		23.1 ± 4.4	
Rural	412.8 ± 74.6		27.8 ± 14.7		23.4 ± 4.9	
County						
County A	408.1 ± 107.8	4.59 (0.204)	18.8 ± 14.4	16.54 (< 0.001)**	23.1 ± 4.4	2.17 (0.538)
County B	412.8 ± 74.6		27.8 ± 14.7		23.4 ± 4.9	
County C	389.7 ± 76.9		20.8 ± 12.8		21.6 ± 5.0	
County D	384.0 ± 95.9		23.3 ± 13.9		21.4 ± 5.2	

Note. *p<.05 **p<.01. LPA Light physical activity, *MVPA* moderate to vigorous physical activity, *FARMS* free and reduced meal services

Teacher race, education level, years of experience, and school FARMS eligibility, racial/ethnic composition, and urbanicity showed no associations with teacher PA promoting practices sum scores and were therefore excluded from the regression models for parsimony.

The unconditional multi-level model yielded a statistically significant estimated school-level variance of 3.65 (*p* = 0.027) as well as a statistically significant estimated residual variance of 21.05 (*p* < 0.001). The ICC was .15, indicating approximately 15% of the total variance of teacher PA promoting practices sum scores was associated with school groupings, justifying the multilevel model.

Table 4 shows results of the multilevel multivariate models for MVPA and teacher PA promoting practices sum scores. The unadjusted model without covariates revealed a significant association between MVPA and teacher PA promoting practices sum score (*p* < 0.001), indicating that for every 15-min increase in MVPA, there was an increase of 1.29 points on the teacher PA promoting practices sum score (*SE* = 0.28). In model 2, after adjusting for individual-level variables, MVPA remained significantly associated with a higher teacher PA promoting practices sum score (coeff = 1.04; *SE* = 0.29, *p* < 0.001), and having an obese weight status (coeff = − 1.42; *SE* = 0.56, *p* = 0.011) was significantly associated with lower scores. In model 3, after adjusting for both individual and school-level factors, teacher MVPA remained significantly associated with a higher teacher PA promoting practices sum score (coeff = 1.07; *SE* = 0.28, *p* < 0.001). Additionally, female gender (coeff = − 1.95; *SE* = .92, *p* = 0.034), having an obese weight status (coeff = − 1.38; *SE* = 0.54, *p* < 0.010), and teaching in a middle school (coeff = − 3.86; *SE* = 0.54, *p* < 0.001) were significantly associated with lower sum scores. The number of days the accelerometer was worn was not significantly associated with teacher PA promoting practices in any of the models.

**Table 4:** Multi-level Multivariate models for MVPA in relation to teacher PA promoting practices sum score

	Teacher PA Promoting Practices Sum Score					
Fixed Effects	Model 1		Model 2		Model 3	
	Estimate + SE	*p*-value	Estimate + SE	*p*-value	Estimate + SE	*p*-value
Teacher MVPA***	**1.29 ± 0.28**	**< 0.001**	**1.04** + 0.29	< 0.001**	**1.07** + 0.28	< 0.001**
Teacher Gender (Female vs. Male)	–	–	−1.62 + 0.94	0.085	**−1.95** + 0.92	0.034*
Teacher Wt. status (Obese vs. non-obese)	–	–	**− 1.42** + 0.56	0.011*	**−1.38** + 0.54	0.010*
School type (Middle vs. Elementary)	–	–	–	–	**−3.86** + 0.54	< 0.001**
**Random Effects**						
	Estimate + SE		Estimate + SE			
Intercept (school)	3.10 ± 1.40	LR test chi(01) = 23.07 p < 0.001	3.00 ± 1.37	LR test chi(01) = 22.03, p < 0.001	< 0.001 + < 0.001	LR test chi(01) < 0.001, *p* = 1.0
Residual	19.66 ± 1.69		19.09 ± 1.64		18.92 + 1.58	

*Note.* *p < .05 **p < .01. Significant regression coefficients are in bold fonts. Model 1 = model with teacher-level variables only. Model 2 = model with teacher- and school-level variable. ***MVPA is represented in 15-min non-contiguous increments of time for this analysis

LPA was not associated with teacher PA promoting practices sum scores before and after adjusting for the covariates (results not shown, but available upon request).

## Discussion

This study sought to identify teacher and school-level factors associated with teacher PA promoting practices and yielded 3 primary findings. First, teachers’ time spent in MVPA was positively associated with their PA promoting practices. Second, teacher-level factors that were negatively associated with teacher PA promoting practices included female gender (versus male) and having an obese weight status (versus non-obese). Third, only teaching middle school (versus elementary) was negatively associated with teacher PA promoting practices, while no associations were found with other school-level factors (e.g., FARMS eligibility, school racial/ethnic composition, and urbanicity). This study advances the current literature on PA in schools by showing that objectively measured teacher PA is associated with teacher PA promoting practices, and contributes to the knowledge-base on the role of teachers and school context in school-based PA. Presented below are implications and future directions for research and practice regarding teacher PA behaviors and their PA promoting practices in schools.

First, using objective measures of PA among teachers, this study demonstrated that personal PA of teachers, specifically time spent in MVPA, is positively associated with their PA promoting practices in and outside of the classroom. Perhaps if teachers are more active, they perceive themselves to be more competent with PA and thus promote more PA promoting practices, as has been found in another study examining classroom PA integration [34]. Many school-based interventions have focused on childhood obesity prevention efforts [47], but few interventions have sought to describe, as we have, health-related behaviors of the school staff, including teachers. In addition to the benefits of participating in PA among teachers for their own mental and physical health, improvement of teacher-level PA may impact their students. Prior studies have shown classroom PA during the school day increased step counts in young children [48]. Moreover, evidence suggests that students engage in more MVPA when they have teachers who value PA [49]. This is particularly important in schools serving lower-income communities located in urban areas, where students are less likely to meet MVPA guidelines [50, 51]. One study found that school-based physical education classes among elementary/middle schools in low-income communities were only providing 23% of the daily MVPA recommendation (60/min/day) [52], thus supplementing with PA promoting practices throughout the school day may increase students’ total PA.

Second, some teacher-specific characteristics were negatively associated with teacher PA promoting practices, including female gender and having an obese weight status. Males had higher teacher PA promoting practices sum scores than females, which is inconsistent with other studies that have shown that gender was not related to implementation of PA promoting practices in the classroom [25]. Years of teaching experience was not associated with teacher PA promoting practices. Prior studies assessing this association have been mixed – one study found that teachers perceived that more experienced colleagues did not implement PA promoting practices because of philosophical differences [30], while another study found that more-experienced teachers tended to implement more PA promoting practices [29]. There is a clear need to conduct more research to determine associations between teachers’ gender, years of experience, their related approaches to teaching, and their PA promoting practices, in order to develop tailored curricula or professional development strategies. Understanding context and needs of schools and teachers may also contribute to the successful development of tailored school PA opportunities [24].

Two variables that are understudied related to teacher PA promoting practices are teacher race and weight status. Although teacher PA promoting practices did not differ by personal race/ethnicity, white teachers had higher MVPA levels. For teacher weight status, associations with teacher PA promoting practices persisted even after accounting for teacher MVPA. Given the clear influential role of teachers on students, it is critical to better understand these associations to implement effective school-based intervention strategies. Future research should use mixed methods strategies to investigate in-depth the mechanisms that may be driving these findings and identify constructs that may be missing from current models (e.g., health knowledge, attitudes, self-efficacy for PA).

Finally, only one school-level factor was associated with teacher PA promoting practices. Teaching in a middle school was associated with less teacher PA promoting practices than elementary school, which is unsurprising, given previously described challenges with implementing PA opportunities with secondary students [15]. Future research should focus on development and testing of interventions that are tailored for the unique needs of secondary school teachers. Furthermore, this study did not identify an association between school FARMS eligibility and teacher PA promoting practices, which was contrary to our hypothesis, as prior literature has shown that lower SES schools have fewer school-wide PA opportunities [35, 36]. Bivariate analyses showed that MVPA significantly differed by school racial/ethnic composition and urbanicity, which is in line with other studies finding that the school PA environment varies by demographic and contextual characteristics [36]. Additionally, MVPA was higher in rural areas, which is surprising given that schools in rural areas tend to be under resourced and offer less PA opportunities for students [35]. Previous research shows that teachers who work in rural schools may be more motivated to positively influence students’ lives and thus take on more tasks or responsibilities to do so [53]. It is possible that this is similarly reflected in our findings. While it is important to keep in mind the additional burdens placed upon teachers in rural or under-resourced schools, their elevated role can potentially be leveraged to improve policies and practices to promote more PA in and outside of the classroom for all students.

### Limitations and strengths

There were several strengths and limitations to this study. A strength of this study is the use of accelerometry to determine LPA and MVPA time to avoid biases associated with self-report measures. The ankle placement of the accelerometer provided continuous 24-h data collection while reducing participant burden (i.e., no need to remove device), but algorithms for this device, placement, and population (adults) have not been developed to distinguish sleep from sedentary time. Another strength of this study is the inclusion of data from teachers within diverse schools, a population often excluded from school-based childhood obesity prevention interventions. Additionally, the sample represented a variety of school characteristics in terms of school level, FARMS eligibility, school racial composition, and urbanicity, and represented 5 distinct school districts. However, data was collected from one state, which may limit generalizability. Furthermore, examining other teacher- and school-level factors not examined in the current study could reveal different insights into factors associated with teacher PA promoting practices. Therefore future research should examine a wider array of variables (e.g., teacher confidence, school physical environment, PA culture and policies, etc.) to understand how to foster teacher engagement and commitment to providing PA opportunities [28]. The self-report nature of the PEAS was also a limitation, as it could have resulted in participants providing socially desirable answers. It is important to note that the study’s cross-sectional design was also a limitation and that the directionality of the relationship between PA and teacher PA promoting practices was not examined. Future research is warranted that examines whether teachers’ personal PA influences the frequency of teacher PA promoting practices they provide, or if teachers’ PA promoting practices result in more teacher PA.

## Conclusions

The results of this study show that teachers who spent more time in daily MVPA also had higher PA promoting practices for their students. In addition, after adjustment for all covariates at the teacher and school-level, only gender, weight status, and school type showed association with teacher PA promoting practices. The results suggest that personal health behaviors may play a role in health promotion behaviors. Strategies that increase PA in schools have the potential to positively impact student PA behaviors; thus, identifying specific target characteristics at both the teacher and school-level is necessary in order to design effective, tailored interventions to promote student-level PA. Future research should examine the direction of the relationship between teachers’ PA behaviors and their PA promoting practices, and perhaps the impact of promoting personal PA to teachers as a way to increase PA opportunities for students.

## Supplementary Information

**Additional file 1.**

## Data Availability

The datasets used and/or analyzed during the current study are available from the corresponding author on reasonable request.

## Abbreviations

CSPAP: Comprehensive school physical activity program
FARMS: free and reduced meal services
ICC: intraclass correlation coefficient
LPA: light physical activity
MVPA: moderate to vigorous physical activity
PA: physical activity
WCC: Wellness Champions for Change
